# Emergency Transport Refusal during the Early Stages of the COVID-19 Pandemic in Gyeonggi Province, South Korea

**DOI:** 10.3390/ijerph19148444

**Published:** 2022-07-11

**Authors:** Min Young Ryu, Hang A. Park, Sangsoo Han, Hye Ji Park, Choung Ah Lee

**Affiliations:** 1 Department of Emergency Medicine, Hallym University, Dongtan Sacred Heart Hospital, 7 Keunjaebong-gil, Hwaseong-si 18450, Korea; alsdudfb12@gmail.com (M.Y.R.); hangapark@hallym.or.kr (H.A.P.); ji4051@hanmail.net (H.J.P.); 2 Department of Emergency Medicine, Soonchunhyang University Bucheon Hospital, Bucheon 14584, Korea; brayden0819@daum.net

**Keywords:** COVID-19, emergency transport, fever, emergency medical services, refusal

## Abstract

We analyzed the changes in patients’ clinical characteristics and transport refusal pre- and post-COVID-19 and identified the reasons for transport refusal using emergency medical services run sheet data from pre-COVID-19 (April–December 2019) and post-COVID-19 (April–December 2020) in Gyeonggi Province, South Korea. We included patients aged ≥18 years. Univariate and multivariate logistic regression analyses were performed to identify the relationship between patients’ personal factors and clinical characteristics and emergency transport refusal. During the control and study periods, 612,681 cases were reported; the transport refusal rates during the control and study periods were 6.7% and 8.2%, respectively. Emergency transport refusal was associated with younger age, the male sex, a normal mental status, a shock index < 1, and trauma in both the pre- and post-COVID-19 periods. Although fever prevented transport refusal during the pre-COVID-19 period (aOR, 0.620; 95% CI, 0.567–0.679), it became a significant risk factor for transport refusal during the post-COVID-19 period (aOR, 1.619; 95% CI, 1.534–1.709). The most common reason for transport refusal by critically ill patients was “because it was not accepted within the jurisdiction and remote transport was required.” It is necessary to expand the response capacity of patients with fever in the community to reduce the refusal of transport by critically ill patients.

## 1. Introduction

Emergency medical services (EMS) are the first link in the chain of healthcare services and the management of public health crises [1]. The coronavirus disease 2019 (COVID-19) pandemic has resulted in a lack of resources and work overload of the EMS and has also affected the health status of patients requiring EMS access through a change in hospital care protocols [2].

As the COVID-19 pandemic progresses, several studies have reported a decline in early EMS use [3,4]. In the early stages of the pandemic, the number of emergency department (ED) visits and the hospitalization rate decreased [4,5,6]. However, it has been reported that transport difficulties have significantly increased [7]. Most EDs strengthened the screening and preemptive isolation of patients with COVID-19-suspected symptoms (fever, respiratory symptoms, and desaturation) through triage [8]. The lack of isolation spaces and the complicated triage process not only increase the prehospital time of the patient but also increase the time spent at the ED before consulting a physician [2] as well as the patient’s refusal of the hospital transport itself [6,8,9].

Patients who refuse to be transferred to the hospital may activate the EMS again or have an increased likelihood of further hospitalization or death [10,11,12]. Therefore, a systematic approach is required to ensure that patients who require hospital treatment can receive appropriate treatment before their physical condition deteriorates.

We aimed to analyze the association between patients’ clinical characteristics and transport refusal pre- and post-COVID-19 and identify the reasons for their transport refusal.

## 2. Materials and Methods

### 2.1. Study Design and Setting

We performed a retrospective analysis to identify refusal of hospital transport pre- and post-COVID-19 using EMS records in Gyeonggi Province, South Korea. According to estimates from 2021, Gyeonggi Province in South Korea covers 10,195 km^2^ with 13.9 million inhabitants [13]. The EMS system is government-based and provides basic-to-intermediate-level EMS from the fire agency headquarters. As of 2021, the Gyeonggi EMS system consists of two headquarters, 35 fire stations with 263 ambulances, and 1912 paramedics [14]. There are 64 EDs in Gyeonggi Province. In principle, patients who develop an emergency within the jurisdiction are transported to a medical institution within the area. If a medical facility in the area cannot accommodate the patient due to a lack of medical staff or hospital beds or is unable to offer specialized treatment, long-distance transport is permitted beyond the jurisdiction.

### 2.2. Participants and Variables

This study compared EMS run sheet data pre-COVID-19 (April to December 2019; control period) and post-COVID-19 (April to December 2020; study period). We included patients aged ≥18 years who were referred to the EMS. Cases of overt death were excluded from the study because transportation to the hospital was not required.

In the EMS run sheet, the patient’s age, sex, main and associated symptoms, initial vital signs, history, mental status, medical categories, treatment history in EMS, and details of patient occurrence were recorded. The shock index (SI) was calculated based on the initial vital signs and a shock index (SI) ≥ 1 was considered a critically ill condition [15,16]. We defined a normal mental status as an alert or verbal response. The medical category was divided into disease and trauma. Fever and respiratory symptoms were considered associated symptoms. Fever was defined as a body temperature ≥ 37.5 °C, and respiratory symptoms were defined as at least one of rhinorrhea, cough, sputum, dyspnea, sore throat, and myalgia. For critically ill patients with a SI ≥ 1, a case analysis was performed on the reasons for refusal of emergency transport. The decision to refuse transport was classified into patient, guardian, and circumstance. Two medical directors with more than 5 years of experience reviewed narrative data to classify the reasons for transport refusal. After consensus on the frequency and importance of reasons, reasons for transport refusal were categorized. The acute management performed by paramedics and the patient’s outcomes were evaluated based on the patient’s condition before the EMS left the patient. Airway management included manual airway manipulation, supraglottic airway, endotracheal intubation, and foreign body removal. Medicine administration included inhalation therapy in addition to intramuscular, intravenous, and oral administration. Trauma management included spinal stabilization, hemostasis, and wound dressing.

### 2.3. Outcomes

The primary outcome was determined as the clinical factors of the patient related to the refusal of transport, and the secondary outcome was the reasons for the transport refusal.

### 2.4. Statistical Analysis

Nominal data were presented as frequencies and percentages, and continuous variables were presented as medians and interquartile ranges (IQR). Categorical variables were compared using the chi-square test or Fisher’s exact test. Nonparametric continuous variables were analyzed using the Mann–Whitney U test. Statistical significance was set at *p* < 0.05. Univariate logistic regression analysis was performed to identify the relationship between patients’ personal factors and clinical characteristics and the refusal of emergency transport. After adjustments for age, sex, medical history, mental status, medical category, initial SI, and associated symptoms, a multivariate logistic regression analysis was performed using the estimated odds ratio (OR) with 95% confidence intervals (CIs). All statistical analyses were performed using the SPSS software (version 25.0; IBM Corp., Armonk, NY, USA).

## 3. Results

**Figure 1:**
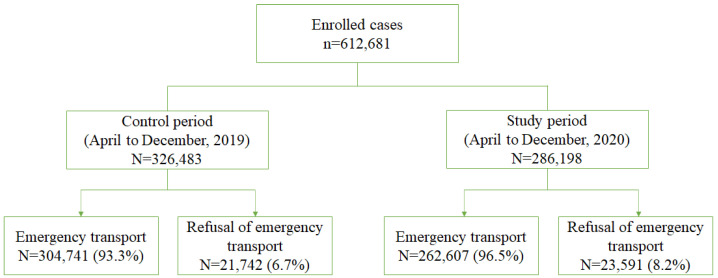
Study flow diagram.

During the control and study periods, a total of 612,681 cases were reported for patients aged >18 years, excluding obvious death. The number of EMS calls between the control and the study periods was 326,483 and 286,198, respectively, which decreased by approximately 12.3%. The refusal rates of transport during each period were 6.7% and 8.2%, respectively, and the refusal rate of emergency transport during the study period increased (Figure 1).

Table 1 shows the general characteristics of the patients who made EMS calls during the study period. The median age of patients who refused emergency transport during the control period was 52 years, which was lower than that of patients who were transported to the hospital. The median age of patients who refused emergency transport was 54 years during the control period. The transport refusal rate increased from 8.1% to 9.4% and 4.8% to 6.6% for men and women, respectively. Refusal of emergency transport increased by 3.3 times for patients with fever and 3.8 times for those with respiratory symptoms.

In the univariate analysis, the refusal rates of emergency transport were higher in the case of a younger age, the male sex, a normal mental status, a SI < 1, a trauma in both the pre- and post-COVID-19 periods, and the underlying causes of hypertension, diabetes mellitus, stroke, liver cirrhosis, and malignancy. Diseases with respiratory or fever symptoms were associated with a higher rate of transport to the hospital. When age, sex, comorbidity, mental status, SI, medical category, and their associated symptoms were adjusted, the refusal rate of emergency transport was significantly lower in the case of fever during the pre-COVID-19 period (aOR, 0.620; 95% CI, 0.567–0.679) and significantly higher during the post-COVID-19 period (aOR, 1.619, 95% CI, 1.534–1.709). Age, sex, comorbidity, mental status, SI, medical category, and respiratory symptom-associated cases did not differ significantly during the pre- and post-COVID-19 periods (Table 2).

Table 3 shows a detailed analysis of critically ill patients with a SI ≥1. The presence or absence of a history of liver cirrhosis during the pre-COVID-19 period did not show a significant association with the refusal of emergency transport (*p* = 0.099) and the presence or absence of a history of stroke post-COVID-19 did not differ significantly from transport refusal (*p* = 0.069).

In cases of transport refusal in critically ill patients, oxygen supplement treatment by paramedics and the administration of fluids or medication significantly increased 4.4 times and 1.8 times during the study. The refusal of emergency transportation due to environmental factors increased by 8.4 times. The number of patients who were not transported to the hospital even though they did not fully recover was 62.2% in the control period but increased to 72.3% in the study period. The decision to refuse transportation due to the surrounding circumstances increased significantly from 2.4% in the control period to 17.4% in the study period. The most common reason for patients’ refusal of transport was “because symptoms have improved or the condition as usual”, and “because it was not accepted within the jurisdiction and remote transport was required” showed the largest increase (Table 4).

## 4. Discussion

Our study is the first to identify the factors associated with the refusal of emergency transport after COVID-19, including the reasons for refusal in critically ill patients in South Korea. Our findings demonstrate that EMS activation decreased after the COVID-19 outbreak but the refusal of emergency transport increased. It was revealed that the refusal occurred more frequently in those who were younger, male, had a SI < 1, had experienced a trauma, and had a fever.

In the early stages of COVID-19, EMS calls tended to decrease. Patients’ fears of spreading an infectious disease caused a rejection of hospital treatment. In addition, with social isolation policies such as social distancing and the avoidance of going out if unnecessary, outside activities and contact with people decreased and infectious diseases such as influenza and trauma decreased [17]. However, patients’ refusal to be transported to hospitals increased.

A refusal of transport was recognized as the patient’s right. According to the standard guidelines for on-site first aid in South Korea, if the patient is an adult > 18 years and is in a sound mental state capable of exercising appropriate judgment, he/she has the right to refuse first aid and transport. Appropriate judgment is the ability to correctly understand the consequences of one’s decisions [18].

The refusal of transport poses a risk to both the patient and paramedics. Previous studies have demonstrated increased rates of repeat requests for EMS, subsequent ED visits, and hospital admission [10,11,12]. Paramedics do not have the right to provide emergency medical care to patients if they refuse emergency treatment on their own. However, when the patient’s condition worsens, paramedics may face legal charges, especially if they are negligent in the process [19]. Increased refusal of transport after COVID-19 could lead to a poor prognosis for patients and lawsuits against paramedics. Therefore, it is important to quickly identify the current status of the refusal of transport and the reasons for its occurrence so that it can be improved.

In this study, as in previous studies, the younger age of the patient was associated with transfer refusal. Although Harrison et al. reported that females showed a higher rate of refusal of transport, there were more male refusals of transport in the present study [6]. In this study, transport refusal was more than five times higher for trauma patients than for other diseases. Since the proportion of male patients with trauma is high, it is presumed that the refusal of transport in male patients is also high [20].

After COVID-19, the symptom of fever appeared to be the most relevant factor in the refusal of transport. Fever has already been reported as a factor that delayed prehospital time during COVID-19 [21]. Most emergency departments require the use of isolation rooms for patients with fever or respiratory symptoms [22,23,24]. Therefore, overcrowding caused by insufficient isolation rooms lengthens the waiting time at the hospital or causes long-distance prehospital transport issues [24]. Patients in isolation rooms often choose to refuse treatment instead of undergoing long waiting times or long-distance transport.

Regarding the refusal of transfer by critically ill patients, there was a significant increase during the COVID-19 period. It is thought that the importance of on-site treatment has increased owing to the increase in prehospital time and the difficulty in selecting a hospital. In particular, interventions for oxygen supply have increased significantly. Treatment in an isolation room is essential for patients with respiratory distress, making it difficult to select a hospital and resulting in long waiting times, which may lead to the abandonment of treatment.

Prior to COVID-19, the decision to refuse transport was primarily based on the patients’ judgment followed by the decision of the guardian. A study by Fan showed that it is typical in non-Western cultures for family members to be given a privileged position in medical decision-making, even for competent individuals [25].

After the COVID-19 outbreak, the number of cases of refusal of transfer on the grounds of “unacceptable in jurisdiction/long-distance transport required” increased significantly. Lack of medical resources and imbalances have led to the transfer of patients to distant hospitals rather than nearby hospitals. Patients often give up on hospital treatment because it is difficult to go to the hospital, whereas children find it difficult to take care of their parents in a distant hospital.

This study has several limitations. First, the patients were evaluated retrospectively based only on the EMS records. Second, this study is a pre/post-study, and the results may not be due to COVID-19 but to changes due to other factors in the year between the two periods. Third, the reasons for the refusal of transport may have been the evaluator’s opinions. Although the characteristics of the patients were objective data, the analysis of transfer failure was based on texts described from the paramedics’ points of view. Fourth, the number of critically ill patients may have been underestimated. There were missing data on the SI since some patients who refused to transfer also refused work-up from the beginning, which resulted in missing values. Finally, after a refusal of transport the patient’s outcome could not be evaluated. Because the EMS record is report-based, two records are developed if the same patient reports twice.

## 5. Conclusions

The factor most associated with a refusal of transport post-COVID-19 compared to pre-COVID-19 was an accompanying fever. Lack of medical resources and imbalance increased patients’ travel distances, leading to them giving up on transport rather than voluntarily refusing to be transported. To provide appropriate treatment it is necessary to improve the system to expand the treatment capacity of patients with fever.

## Figures and Tables

**Table 1:** General characteristics of patients with EMS activation pre- and post-COVID-19.

	Control Period		Study Period		
	Transport N = 304,741 (93.3)	Refusal of Transport N = 21,742 (6.7)	Transport N = 262,607 (96.5)	Refusal of Transport N = 23,591 (8.2)	*p*-Value
**Age, year**	58 (43–74)	52 (68–64)	59 (43–74)	54 (38–66)	<0.001
Sex *					
Male	159,855 (91.9)	14,164 (8.1)	140,512 (90.6)	14,567 (9.4)	<0.001
Female	144,841 (95.2)	7333 (4.8)	122,092 (93.4)	8691 (6.6)	<0.001
Mental status *					
Normal mental status	290,190 (94.5)	16,853 (5.5)	247,412 (92.4)	20,211 (7.6)	<0.001
Altered mental status	14,551 (99.1)	129 (0.9)	15,194 (98.2)	277 (1.8)	<0.001
SI *					
≥1.0	152,11 (97.6)	374 (2.4)	13,688 (96.5)	495 (3.5)	<0.001
<1.0	281,338 (94.4)	16,543 (5.6)	240,000 (92.3)	19,908 (7.7)	<0.001
**Comorbidity**					
Hypertension	91,451 (98.0)	1864 (2.0)	80,370 (96.8)	2648 (3.2)	<0.001
Diabetes mellitus	53,246 (97.3)	1498 (2.7)	47,741 (96.1)	1948 (3.9)	<0.001
Stroke	17,094 (97.7)	409 (2.3)	15,562 (96.7)	532 (3.3)	<0.001
Liver cirrhosis	3778 (96.8)	124 (3.2)	3337 (96.1)	135 (3.9)	0.100
Malignancy	17,585 (98.2)	322 (1.8)	16,475 (96.9)	519 (3.1)	<0.001
Medical category *					
Disease	195,041 (97.5)	5001 (2.5)	169,958 (96.8)	5679 (3.2)	<0.001
Trauma	108,890 (87.1)	16,156 (12.9)	92,032 (84.0)	17,489 (16.0)	<0.001
**Associated symptoms**					
Fever	33,961 (98.1)	656 (1.9)	32,965 (93.7)	2222 (6.3)	<0.001
Respiratory symptoms	28,041 (99.4)	177 (0.6)	36,104 (97.7)	858 (2.3)	<0.001

Data are presented as medians (interquartile ranges) or numbers (%). SI, shock index. * Sex, mental status, medical category, and shock index have missing values for 626, 7864, 2435, and 25,124 cases, respectively.

**Table 2:** Unadjusted and adjusted comparisons of refusal rates for EMS transport between the control period and study period.

	Control Period		Study Period	
	OR (95% CI)	aOR (95% CI)	OR (95% CI)	aOR (95% CI)
**Age, year**	1.016 (1.015–1.017)	1.002 (1.001–1.003)	1.012 (1.012–1.013)	1.006 (1.005–1.007)
**Sex, male**	1.750 (1.700–1.802)	1.444 (1.392–1.498)	1.456 (1.417–1.497)	1.248 (1.209–1.289)
**Normal mental status**	6.551 (5.505–7.796)	3.261 (2.706–3.93)	4.481 (3.975–5.051)	2.196 (1.932–2.495)
**SI < 1.0**	2.392 (2.156–2.653)	1.370 (1.222–1.535)	2.294 (2.095–2.512)	1.420 (1.287–1.566)
**Comorbidity**				
Hypertension	0.219 (0.208–0.229)	0.386 (0.364–0.409)	0.287 (0.275–0.299)	0.413 (0.394–0.434)
Diabetes mellitus	0.350 (0.331–0.369)	0.862 (0.810–0.918)	0.405 (0.386–0.425)	0.820 (0.777–0.865)
Stroke	0.323 (0.292–0.356)	0.743 (0.665–0.830)	0.366 (0.336–0.400)	0.713 (0.649 - 0.784)
Liver cirrhosis	0.457 (0.382–0.547)	0.849 (0.697–1.033)	0.447 (0.376–0.531)	0.781 (0.650–0.938)
Malignancy	0.245 (0.220–0.274)	0.552 (0.488–0.624)	0.336 (0.308–0.367)	0.616 (0.559–0.678)
**Disease**				
**Medical category, trauma**	5.786 (5.601–5.978)	2.754 (2.651–2.862)	5.687 (5.514–5.866)	3.547 (3.425–3.675)
**Associated symptoms**				
fever	0.248 (0.229–0.268)	0.620 (0.567–0.679)	0.724 (0.692–0.758)	1.619 (1.534–1.709)
respiratory symptom	0.081 (0.070–0.094)	0.278 (0.237–0.327)	0.237 (0.221–0.254)	0.529 (0.488–0.573)

OR, odds ratio; CI, confidential interval; aOR, adjusted odds ratio, SI, shock index.

**Table 3:** Characteristics of patients who failed to use EMS transport with a shock index ≥ 1.0.

	Control Period N = 15,585			Study Period N = 14,183		
	Transfer N = 15,211	No Transfer N = 374	*p*-Value	Transfer N = 13,688	No Transfer N= 495	*p*-Value
**Age, year**	59 (41–77)	50 (32–65)	<0.001	61 (43–78)	53 (35–71)	<0.001
**Sex**			<0.001			0.014
Male	8124 (97.2)	233 (2.8)		7304 (96.2)	288 (3.8)	
Female	7087 (98.1)	138 (1.9)		6383 (97.0)	200 (3.0)	
**Normal mental status**	13,491 (97.7)	319 (2.3)	<0.001	11,825 (96.4)	444 (3.6)	<0.001
**Decreased mental status**	1720 (99.7)	5 (0.3)		1863 (98.9)	20 (1.1)	
**Comorbidity**						
Hypertension	3922 (98.9)	44 (1.1)	<0.001	3789 (98.2)	68 (1.8)	<0.001
Diabetes mellitus	2948 (98.6)	43 (1.4)	<0.001	2996 (98.3)	52 (1.7)	<0.001
Stroke	1238 (98.8)	15 (1.2)	0.004	1125 (97.5)	29 (2.5)	0.069
Liver cirrhosis	492 (98.8)	6 (1.2)	0.099	408 (98.3)	7 (1.7)	0.041
Malignancy	2020 (99.2)	17 (0.8)	<0.001	2015 (98.2)	36 (1.8)	<0.001
**Medical category**						
Disease	12,481 (98.7)	170 (1.3)	<0.001	11,454 (98.1)	218 (1.9)	<0.001
Trauma	2718 (93.1)	201 (6.9)		2216 (89.1)	272 (10.9)	
**Associated symptoms**						
fever	4389 (98.5)	68 (1.5)	<0.001	4700 (97.0)	145 (3.0)	0.021
respiratory symptom	4382 (99.6)	16 (0.4)	<0.001	5736 (98.6)	80 (1.4)	<0.001

Data are presented as medians (interquartile ranges) or numbers (%).

**Table 4:** Case analysis of transport failure in critically ill patients.

	Control Period N = 374	Study Period N = 495	*p*-Value
**Prehospital intervention by paramedics**			0.033
No	271 (72.5)	325 (65.7)	
Yes	103 (27.5)	170 (34.3)	
Airway management	33 (8.8)	45 (9.1)	0.905
Oxygen supplement	9 (2.4)	52 (10.5)	<0.001
Administration of fluids or medication	35 (9.4)	83 (16.8)	0.002
Trauma management	43 (11.5)	54 (10.9)	0.828
**Decision of transport refusal**			<0.001
By patient	329 (88.0)	349 (70.5)	
By Caregivers	36 (9.6)	60 (12.1)	
By circumstantial factors	9 (2.4)	86 (17.4)	
**Reason for transport refusal**			<0.001
Improvement/No change from usual	325 (86.9)	365 (73.7)	
Private transport for wanted hospital	10 (2.7)	27 (5.5)	
Unacceptable in jurisdiction/long-distance transport required	1 (0.3)	80 (16.2)	
Economic problem	7 (1.9)	7 (1.4)	
Unable to be assessed as violent behavior caused by drinking alcohol	31 (8.3)	16 (3.2)	
**Outcome**			
Complete improvement	119 (31.8)	132 (26.7)	0.012
Partial improvement	67 (17.9)	65 (13.1)	
No change	188 (50.3)	298 (60.2)	

Data are presented as numbers (%).

## Data Availability

Data used to support the findings of this study are available from the corresponding author upon request.

## References

[1] Mohammadi F., Tehranineshat B., Bijani M., Khaleghi A.A. (2021). Management of COVID-19-related Challenges Faced by EMS Personnel: A Qualitative Study. BMC Emerg. Med..

[2] Kim D., Jung W., Yu J.Y., Chang H., Lee S.U., Kim T., Hwang S.Y., Yoon H., Shin T.G., Sim M.S. (2022). Effect of Fever or Respiratory Symptoms on Leaving Without Being Seen During the COVID-19 Pandemic in South Korea. Clin. Exp. Emerg. Med..

[3] Venkatesh A.K., Janke A.T., Shu-Xia L., Rothenberg C., Goyal P., Terry A., Lin M. (2021). Emergency Department Utilization for Emergency Conditions During COVID-19. Ann. Emerg. Med..

[4] Handberry M., Bull-Otterson L., Dai M., Mann N.C., Chaney E., Ratto J., Horiuchi K., Siza C., Kulkarni A., Gundlapalli A.V. (2021). Changes in Emergency Medical Services Before and During the COVID-19 Pandemic in the United States, January 2018–December 2020. Clin. Infect. Dis..

[5] Dezman Z.D.W., Stryckman B., Zachrison K.S., Conrad R.M., Marcozzi D., Pimentel L., Samuels-Kalow M., Cairns C.B. (2021). Masking for COVID-19 Is Associated with Decreased Emergency Department Utilization for Non-COVID Viral Illnesses and Respiratory Conditions in Maryland. Am. J. Med..

[6] Harrison N.E., Ehrman R.R., Curtin A., Gorelick D., Hill A.B., Brennan E., Dunne R. (2021). Factors Associated with Voluntary Refusal of Emergency Medical System Transport for Emergency Care in Detroit During the Early Phase of the COVID-19 Pandemic. JAMA Netw. Open..

[7] Igarashi Y., Yabuki M., Norii T., Yokobori S., Yokota H. (2021). Quantitative Analysis of the Impact of COVID-19 on the Emergency Medical Services System in Tokyo. Acute Med. Surg..

[8] Satty T., Ramgopal S., Elmer J., Mosesso V.N., Martin-Gill C. (2021). EMS Responses and Non-transports During the COVID-19 Pandemic. Am. J. Emerg. Med..

[9] Siman-Tov M., Strugo R., Podolsky T., Blushtein O. (2021). An Assessment of Treatment, Transport, and Refusal Incidence in a National EMS’s Routine Work During COVID-19. Am. J. Emerg. Med..

[10] Moss S.T., Chan T.C., Buchanan J., Dunford J.V., Vilke G.M. (1998). Outcome Study of Prehospital Patients Signed Out Against Medical Advice by Field Paramedics. Ann. Emerg. Med..

[11] Burstein J.L., Henry M.C., Alicandro J., Gentile D., Thode H.C., Hollander J.E. (1996). Outcome of Patients Who Refused Out-of-Hospital Medical Assistance. Am. J. Emerg. Med..

[12] Knight S., Olson L.M., Cook L.J., Mann N.C., Corneli H.M., Dean J.M. (2003). Against All Advice: An Analysis of Out-of-Hospital Refusals of Care. Ann. Emerg. Med..

[13] (2022). Gyeonggi-do. 2021 Gyeonggi-do Resident Registration Demographics; Gyeonggi-do. https://stat.gg.go.kr/statgg/kr/dataMng/PublicationForm.html?pub_seq=2.

[14] National Fire Agency (2021). Statistical Yearbook 2020 of 119 Emergency Services.

[15] Jouffroy R., Pierre Tourtier J., Gueye P., Bloch-Laine E., Bounes V., Debaty G., Boularan J., Carli P., Vivien B. (2020). Prehospital Shock Index to Assess 28-Day Mortality for Septic Shock. Am. J. Emerg. Med..

[16] Jehan F., Con J., McIntyre M., Khan M., Azim A., Prabhakaran K., Latifi R. (2019). Pre-hospital shock index correlates with transfusion, resource utilization and mortality; The role of patient first vitals. Am. J. Surg..

[17] O’Connor A.W., Hannah H.A., Burnor E.A., Fukutaki K.G., Peterson T., Ballard D.W., Ereman R.R., Willis M.D., Augusto O.J., Wagenaar B.H. (2021). Emergency Medical Service Utilization and Response Following COVID-19 Emergency and Stay-at-Home Policies: An Interrupted Time-Series Analysis. Cureus.

[18] Kang D.H., Kim R.A., Kim J.C., Kim M.S., Lee I.J. (2019). Guidelines for First Aid for 119 Paramedics.

[19] Bae H.A., Lee S.J., Kim C.W., Lee K.H. (2005). Legal Consideration of Transfer Refusal by 119 Rescuers. Fire Sci. Eng..

[20] Shah M.N., Bazarian J.J., Mattingly A.M., Davis E.A., Schneider S.M. (2004). Patients with head injuries refusing emergency medical services transport. Brain Inj..

[21] Kim H.S., Jang T.C., Kim G.M., Lee S.H., Ko S.H., Seo Y.W. (2020). Impact of the Coronavirus Disease 2019 Outbreak on the Transportation of Patients Requiring Emergency Care. Medicine.

[22] Kim S.C., Kong S.Y., Park G.J., Lee J.H., Lee J.K., Lee M.S., Han H.S. (2021). Effectiveness of negative pressure isolation stretcher and rooms for SARS-CoV-2 nosocomial infection control and maintenance of South Korean emergency department capacity. Am. J. Emerg. Med..

[23] Pantazopoulos I., Tsikrika S., Kolokytha S., Manos E., Porpodis K. (2021). Management of COVID-19 Patients in the Emergency Department. J. Pers. Med..

[24] Park H.A., Kim S., Ha S.O., Han S., Lee C. (2022). Effect of Designating Emergency Medical Centers for Critical Care on Emergency Medical Service Systems During the COVID-19 Pandemic: A Retrospective Observational Study. J. Clin. Med..

[25] Fan R. (2015). Family-Oriented Informed Consent: East Asian and American Perspectives (Philosophy and Medicine Book 121).

